# Identifying high snakebite risk area under climate change for community education and antivenom distribution

**DOI:** 10.1038/s41598-023-35314-1

**Published:** 2023-05-20

**Authors:** Masoud Yousefi, Saeed Hosseinian Yousefkhani, Marc Grünig, Anooshe Kafash, Mahdi Rajabizadeh, Eskandar Rastegar Pouyani

**Affiliations:** 1 Department of Animal Science, School of Biology, Damghan University, Damghan, Iran; 2 LIB, Museum Koenig, Bonn, Leibniz Institute for the Analysis of Biodiversity Change, Adenauerallee 127, 53113 Bonn, Germany; 3 Ecosystem Dynamics and Forest Management Group, School of Life Sciences, Technical University of Munich (TUM), 85354 Freising, Germany; 4 Department of Environmental Science, Faculty of Natural Resources, University of Tehran, Karaj, Iran; 5 Department of Biodiversity, Institute of Science and High Technology and Environmental Sciences, Graduate University of Advanced Technology, Kerman, 7631133131 Iran; 6 AI.Nature Team, INRIA Startup Studio, 2 Rue Simone IFF, 75012 Paris, France; 7 Department of Biology, Faculty of Science, Hakim Sabzevari University, Sabzevar, Iran

**Keywords:** Ecological epidemiology, Ecological modelling, Climate change, Public health

## Abstract

Snakebite is one of the largest risks from wildlife, however little is known about venomous snake distribution, spatial variation in snakebite risk, potential changes in snakebite risk pattern due to climate change, and vulnerable human population. As a consequence, management and prevention of snakebite is hampered by this lack of information. Here we used habitat suitability modeling for 10 medically important venomous snakes to identify high snakebite risk area under climate change in Iran. We identified areas with high snakebite risk in Iran and showed that snakebite risk will increase in some parts of the country. Our results also revealed that mountainous areas (Zagros, Alborz, Kopet–Dagh mountains) will experience highest changes in species composition. We underline that in order to improve snakebite management, areas which were identified with high snakebite risk in Iran need to be prioritized for the distribution of antivenom medication and awareness rising programs among vulnerable human population.

## Introduction

Snakebite envenoming is a medical emergency and an important health problem worldwide^1–3^. Up to 5 million snakebites occur each year leading to about 100,000 deaths annualy ^2,4–6^. Despite recent attention to the challenge of snakebite ^3,5,7–10^ little is known about the distribution of venomous snakes, and therefore the spatial variation in snakebite risk and accessibility of vulnerable human population to healthcare system^3^. Further, the potential shift of high risk areas for snakebite due to climate change remains unknown. As a consequence, management of snakebite is hampered by this lack of information around the globe^3,11^. To ensure successful snakebite management it is necessary to identify high snakebite risk areas.

Identifying areas with high risk of snakebite can help us to locate target areas for awareness raising programs and to effectively provide antivenoms to the most vulnerable groups^2,12,13^. While our knowledge on current snakebite risk patterns remains limited^2,14^, venomous snakes are responding to climate change by shifting their habitats to cope with global warming^2,15–17^. Thus, the complexity of snakebite management further increases and therefore calls for more efforts to identify high snakebite risk areas under climate change^2,10^.

Habitat Suitability Models (HSMs) are practical tools to predict the impacts of climate and land use changes on species distribution^18–20^, identify their future distribution^21^, document changes in species composition^22,23^ by correlating species occurrence records to climatic variables^18^. Moreover, HSMs can be used to predict changes in distribution and interaction of species and their host or prey and predator in changing climate^24–26^. In recent years, HSMs are used in predicting snakebite risk areas under current and future climate change, identifying vulnerable human population^2,13,27–30^, and have even been shown to correlate with snakebite incidences^28^. In these studies, venomous snakes’ habitat suitability was considered as an indicator of snakebite risk^2,13,27^.

Iran is home to 15 terrestrial venomous snakes including one of the most famous snake in the world named Spider tailed viper, *Pseudocerastes urarachnoides*^31^. While all 15 species are medically important, four species (*Echis carinatus Macrovipera lebetina*, *Naja oxiana*, and *P. persicus*) are more widespread in Iran and are therefore responsible for the most snakebite incidents in the country^32^. Although previous studies investigated the challenge of snakebite in Iran by reporting snakebite incidents^32,33^ still little is known about medically important venomous snakes’ distribution and potential changes in their distribution under climate change^17,34–36^. Previous studies have shown that climate change will change distribution pattern of biodiversity including snakes in Iran^37^. While suitable habitats of some species will expand under climate change some others will lose considerable proportion of their suitable range. For instance, Yousefi et al.^17^ showed that mountains vipers of genus *Montivipera* will lose their suitable habitat and will shift to higher altitude to track their suitable climate. As model projections showed that species like *E. carinatus* and *P. persicus* will experience an expansion of suitable habitat area due to warming climate, this may lead to an increasing number of envenoming events^38^.

In order to mitigate the risk of snakebite and to identify vulnarable human populations, developing snakebite risk maps becomes crucial^2^. Thus, to effectively manage snakebite problem in Iran the aims of this study are (i) modeling the habitat suitability of medically important venomous snakes; (ii) determining hotspots of snakebite risk; (iii) quantifying changes in medically important venomous snakes’ composition due to climate change; (iv) identifying vulnerable populations to snakebite envenoming by considering exposure to snakebite and accessibility to health centers. The results of this study will guide responsible health organizations for the distribution of antivenom medication and awareness raising programs in the country.

## Results

**Figure 1:**
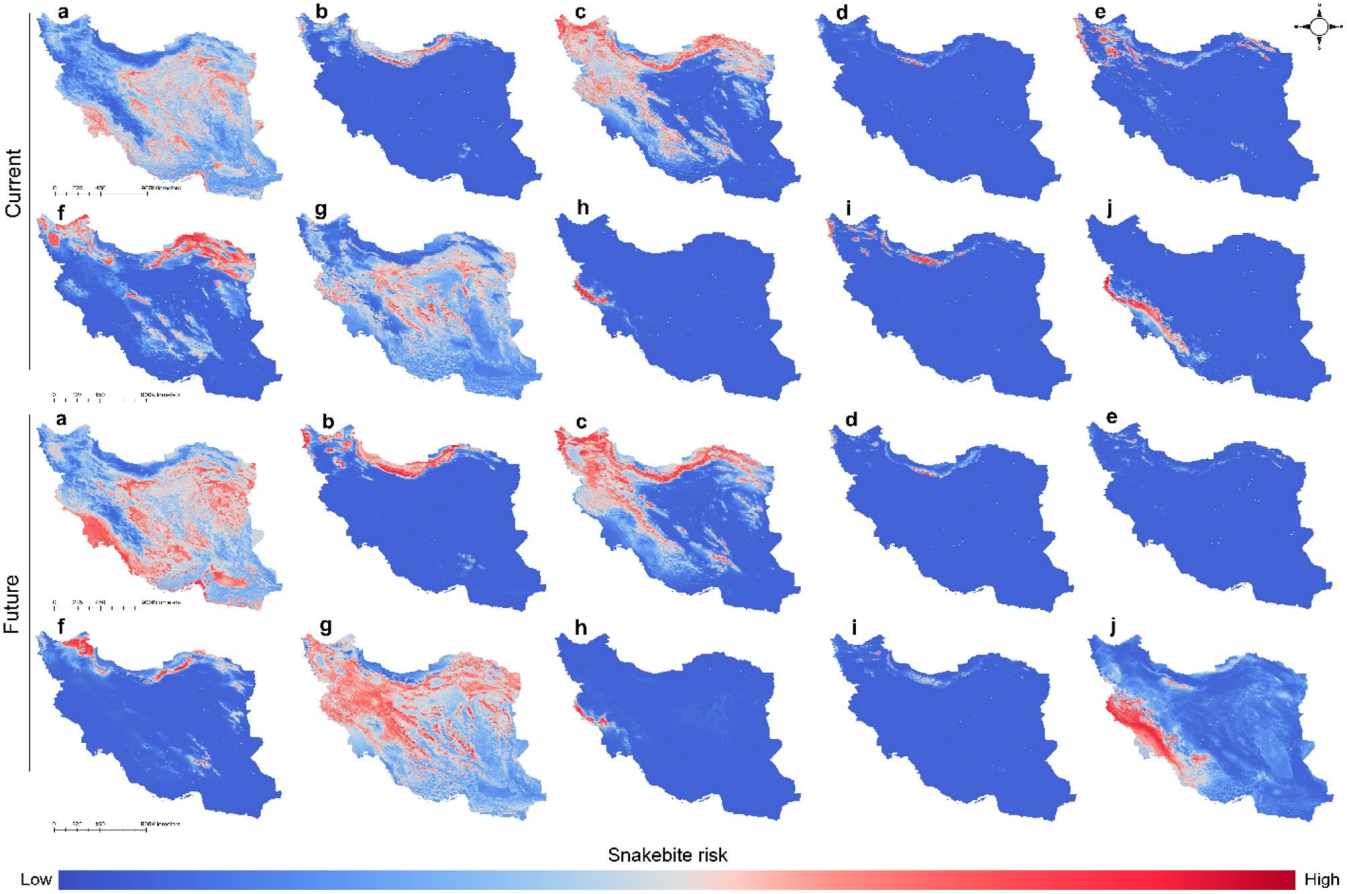
Snakebite risk maps of the 10 species (*Echis carinatus* (**a**), *Gloydius halys* (**b**), *Macrovipera lebetina* (**c**), *Montivipera latifii* (**d**), *Montivipera raddei* (**e**), *Naja oxiana* (**f**), *Pseudocerastes persicus* (**g**), *Pseudocerastes urarachnoides* (**h**), *Vipera eriwanensis* (**i**), *Walterinnesia aegyptia* (**j**)) under current and future (year 2071–2100 SSP585) climatic. Maps were generated using QGIS 3.4.1 (https://www.qgis.org).

**Figure 2:**
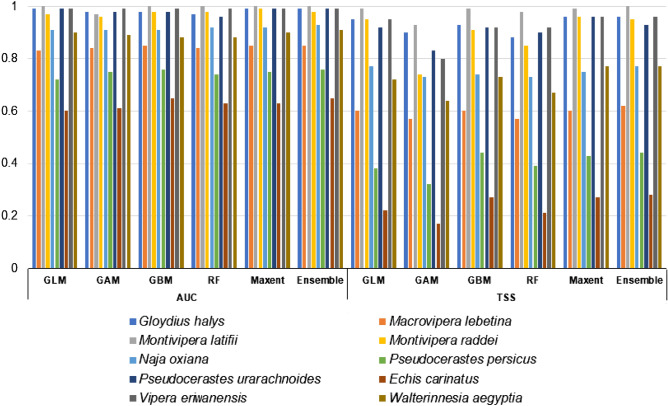
Results of the evaluation metrics (AUC and TSS) of the 10 medically important snakes’ models.

Modeling the impacts of climate change (years 2041–2070 SSP126 (Fig. S1), SSP585 (Fig. S2) and 2071–2100 SSP126 (Fig. S3), SSP585 (Fig. 1)) on habitat suitability of the 10 medically important venomous snakes showed that suitable habitats of five species (*E. carinatus*, *Gloydius halys*, *Montivipera latifii*, *P. persicus* and *Walterinnesia aegyptia*) will increase while five species will lose (*M. lebetina*, *M*. *raddei*, *N. oxiana*, *P. urarachnoides* and *Vipera eriwanensis*) some of their suitable habitat under climate (Fig. 1). Results of the evaluation metrics of the models were presented in Fig. 2. The evaluation metrics values were highest for *M. latifii* while the values were lowest for the *E. carinatus*.

### Variable importance

**Figure 3:**
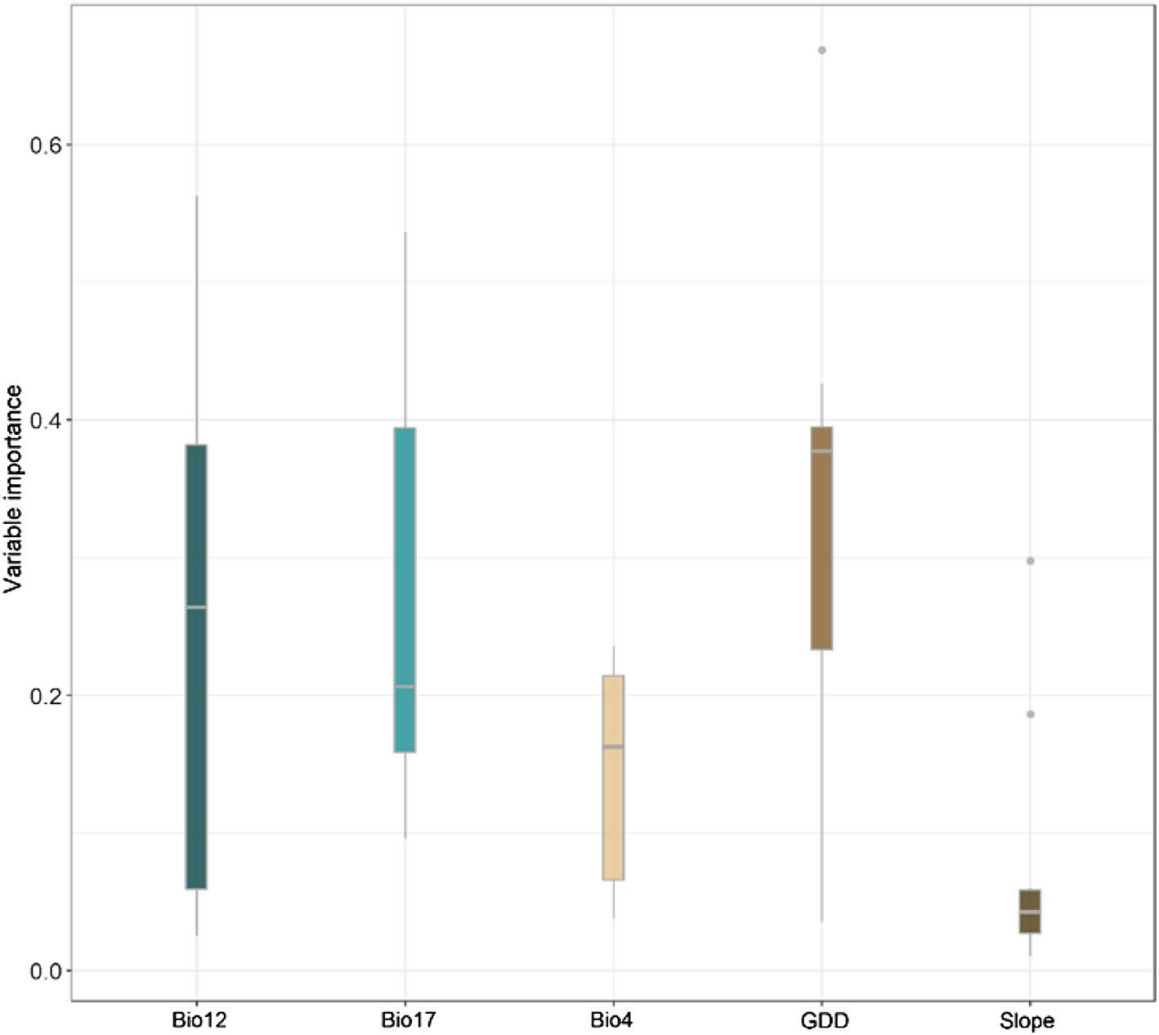
Boxplot of the variables (annual precipitation (Bio12), precipitation of the driest quarter (Bio17), temperature seasonality (Bio4), growing degree days heat sum above 10 °C (GDD) and slope) importance averaged across the 12 species. Boxplot was created in R environment (https://cran.r-project.org).

Estimating variable importance for the 10 medically important venomous snakes showed that growing degree days heat sum above 10 °C followed by annual precipitation were the most important predictor of habitat suitability of the 10 snakes when averaged across all species (Fig. 3). Growing degree days heat sum above 10 °C was the most important determinants of suitable habitats of *M. raddei*, *N. oxiana*, *P. persicus* and *V. eriwanensis*, annual precipitation was identified the most influential predictor of habitat suitability for *E. carinatus*, *M. lebetina* and *W. aegyptia* and precipitation of driest quarter turn out to be the most important predictor of habitat suitability of *G. halys*, *M. latifii* and *P. urarachnoides*.

### Hotspot of snakebite in Iran

**Figure 4:**
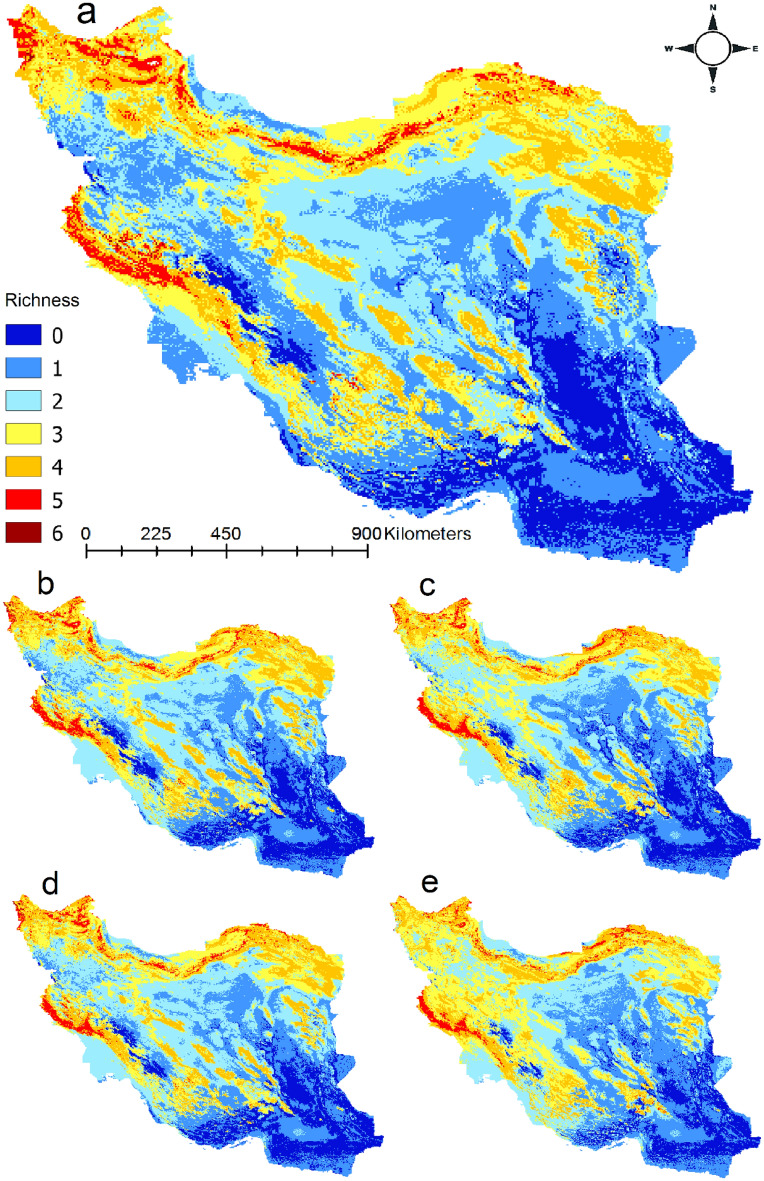
Hotspots of snakebite risk by multiplying habitat suitability models of the 10 medically important venomous snakes in Iran under current (**a**) and future climatic conditions (2041–2070 SSP126 (**b**), 2041–2070 SSP585 (**c**), 2071–2100 SSP126 (**d**), 2041–2070 SSP585 (**e**)). Maps were generated using QGIS 3.4.1 (https://www.qgis.org).

By multiplying snakebite risk models of the 10 medically important venomous snakes (Fig. 4) we found that hotspots of snakebite in Iran are located in north west of Iran, Alborz mountains, and west of Zagros mountains. Zagros mountains have low richness of medically important venomous snakes under current climatic conditions but under climate change snakebite risk will increase in the mountains in particular under SSP585 scenario for years 2071–2100.

### Species composition change

**Figure 5:**
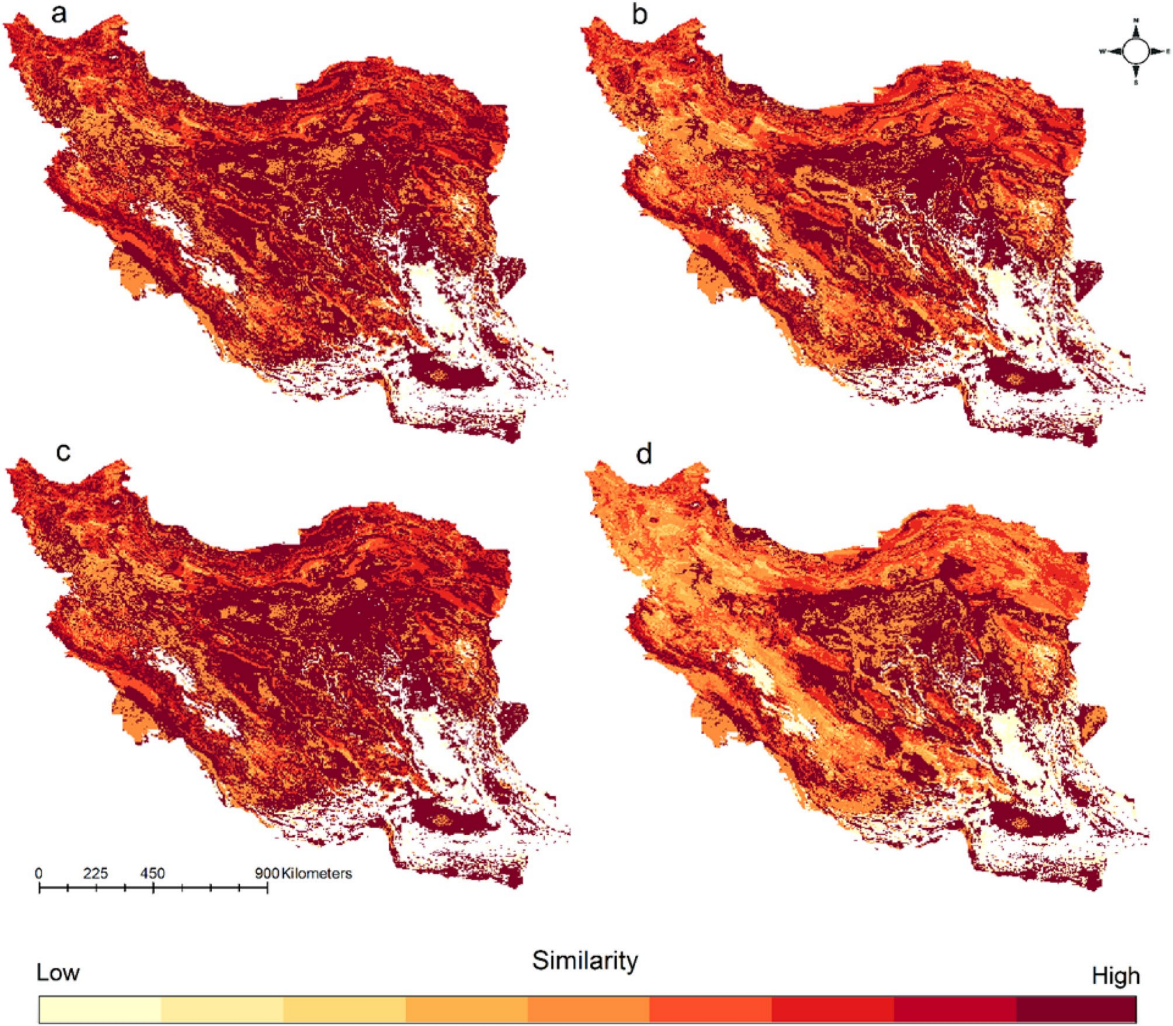
Similarity maps showing change in species composition by comparing current and future distribution (2041–2070 SSP126 (**a**), 2041–2070 SSP585 (**b**), 2071–2100 SSP126 (**c**), 2041–2070 SSP585 (**d**)) of the 10 medically important venomous snakes. Maps were generated using QGIS 3.4.1 (https://www.qgis.org).

We compared similarity of medically important venomous snakes’ composition by comparing current and future distribution of the 10 medically important venomous snakes (Fig. 5). We found that under SSP585 for years 2041–2070 and 2071–2100 mountainous areas (Zagros, Alborz, Kopet–Dagh mountains) will experience highest changes in species composition.

### Vulnerability to snakebite

**Figure 6:**
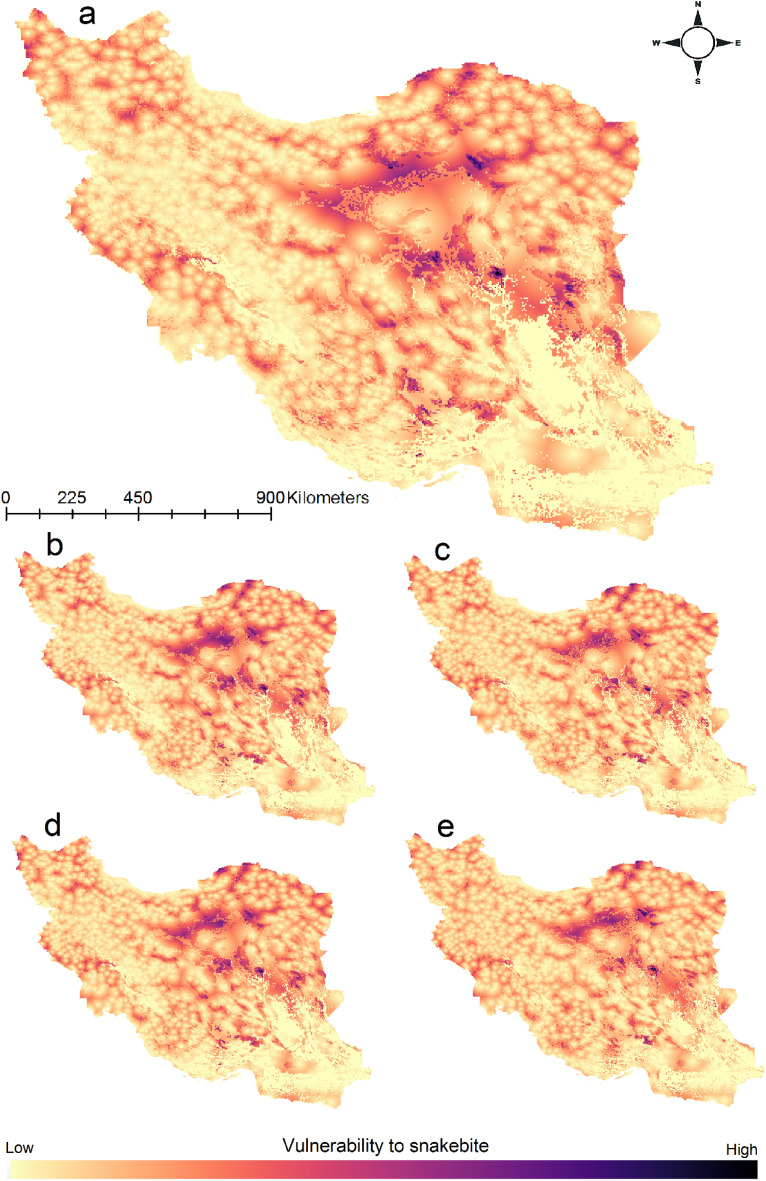
Vulnerability to snakebite maps (exposure to snakebite (the 10 medically important venomous snakes) plus accessibility to healthcare centers), current (**a**), 2041–2070 SSP126 (**b**), 2041–2070 SSP585 (**c**), 2071–2100 SSP126 (**d**), 2071–2100 SSP585 (**e**). Maps were generated using QGIS 3.4.1 (https://www.qgis.org).

Vulnerability to snakebite was modeled by considering exposure to snakebite plus accessibility to healthcare centers. Results showed that central parts of Iran and north east of the country have largest areas which are vulnerable to snakebite. Under climate change geographic pattern of snakebite risk will change especially in Zagros Mountains and areas vulnerable to snakebite will increase (Fig. 6).

## Discussion

Snakebite is a neglected tropical disease and important health problem^39^, leading to thousands of fatal incidents and many more disabilities annually. Hereby, the rural population is at a higher risk of snakebite due to limited access to healthcare facilities, resulting in delayed administration of antivenom, which is a critical factor in the treatment of snakebite^14,40^. To ensure successful snakebite management and reduce snakebite incidents, identifying high snakebite risk areas under current and future climate is highly relevant. Here we mapped areas with high risk snakebite in Iran under current and future climate based on 10 medically important venomous snakes.

We found that the North west of Iran, Alborz mountains, and west of Zagros mountains have the highest richness of medically important venomous snakes and thus are regions with high snakebite risk. Our study confirms the results of previous studies on the importance of climate on distribution of venomous snakes of Iran^13,15,34,38^. Further, we showed that growing degree days heat sum above 10 °C followed by annual precipitation were the most important predictors of habitat suitability of the 10 snakes in Iran. Growing degree days heat sum above 10 °C is the most important variable because it influences the growth and development of plants and insects during the growing season which directly and indirectly influence prey availability for the snakes^41^.

Developing snakebite management strategies based solely on the current distribution of medically significant venomous snake species may be risky, as recent studies have demonstrated that climate change is capable of altering the potential distribution of these species^10,29^. Furthermore, model predictions suggest that climate change may lead to changes in the composition and species richness of medically significant venomous snake species, which could subsequently modify the pattern of snakebite risk^10,29^. Predicted changes in medically important venomous snakes’ hotspot and composition in mountains regions of Iran may result in previously less affected populations to venomous snakes becoming more exposed. The future snakebite risk models developed in this study can be useful to raise awareness among the people who might be at risk of envenoming under climate change and by considering potential changes in snakebite risk pattern for antivenom distribution in the future. Together, this may support reducing the numbers of deaths and cases of disability due to snakebite envenoming^2,10,42^.

Access to antivenom can help to reduce mortality and morbidity of snakebite envenoming^42^. Mapping medically important venomous snakes’ current and future distribution is critical for knowing where antivenin for each species is needed^2^. Beside determining areas with less accessibility to medical facilities is necessary to identify the most vulnerable populations^2,42^. Our models, showing the most vulnerable areas to snakebite in Iran can guide antivenom distribution by the Ministry of Health and Medical Education across the country.

WHO introduced community empowerment and engagement as an important strategy to prevent and control snakebite envenoming^39^. Areas identified at risk of snakebite have high priority to guide snakebite mitigation and training measures for health teams and local people in Iran^2^. Farmers, shepherd and nomads who encounter snakes in their everyday life while working on farms or grazing their livestock are the most vulnerable groups. Thus, awareness raising programs should focus on these population groups. In most snakebite accidents the snakes were not identified and therefore no specific treatment can be applied. Educating local communities in areas with high snakebite risk to be able to identify venomous snakes should therefore be prioritized^40^. Moreover, despite the importance of the snakebite in Iran still health workers are not familiar with all medically important venomous snakes of Iran. Thus, beside local people, it is necessary to regularly educate health workers in the country. Some occupations are associated with higher risk of encountering venomous snakes such as protected areas rangers, foresters and tour guides. Thus, organizations responsible for these occupations like Department of Environment and Department of Natural Resources and Watershed Management can run awareness raising and training workshops in high snakebite risk areas to educate them how to avoid snakebite and to seek health care instead seeking care from traditional healers^39^.

Venomous snakes have secretive behavior and life history thus studying snakebite risk is a challenging task especially in developing countries like Iran^43,44^. Using citizen science is a promising avenue to overcome lack of knowledge on distribution of venomous snake^43^. We encourage using citizen science in Iran as increasing number of young people are getting interested in herping in the country. Their observations on venomous snakes’ distribution in areas with limited data can be used to develop high resolution distribution maps and more accurate models. To be able to use herpers observations in snakes and snakebite studies their observations should be documented with a photograph and deposited in relevant databases like iNaturalist or HerpMapper^43^.

## Conclusions

Identifying high snakebite risk areas under climate change, distribution of effective antivenoms, and education are four key factors in snakebite management. In this study, we predicted the impacts of climate change on 10 medically important venomous snakes, their richness, community composition and identified vulnerable snakebite area. This research study has important implications for snakebite management in Iran. As shown in other studies^13,27–30,45,46^ and confirmed in this research HSMs developed based on distribution of medically important venomous snakes can guide public policies for the distribution of antivenom medication and identification of regions with higher snakebite risk to guide mitigation and training measures for vulnerable populations as well as health teams. It should be noted that population density, educational level, income, etc. could are also important variables that affect people’s living standards and their exposure to snake habitat proximity. But since these predictors are not available at high spatial resolution, we were unable to consider them. Apart from Iran, snakebite is an important challenge in other Middle Eastern countries^32,47^. Our approach in modeling snakebite risk and mapping vulnerability to snakebite under current and future climate can be applied in other countries to reduce snakebite incidents in the region.

## Methods

### Species data

**Figure 7:**
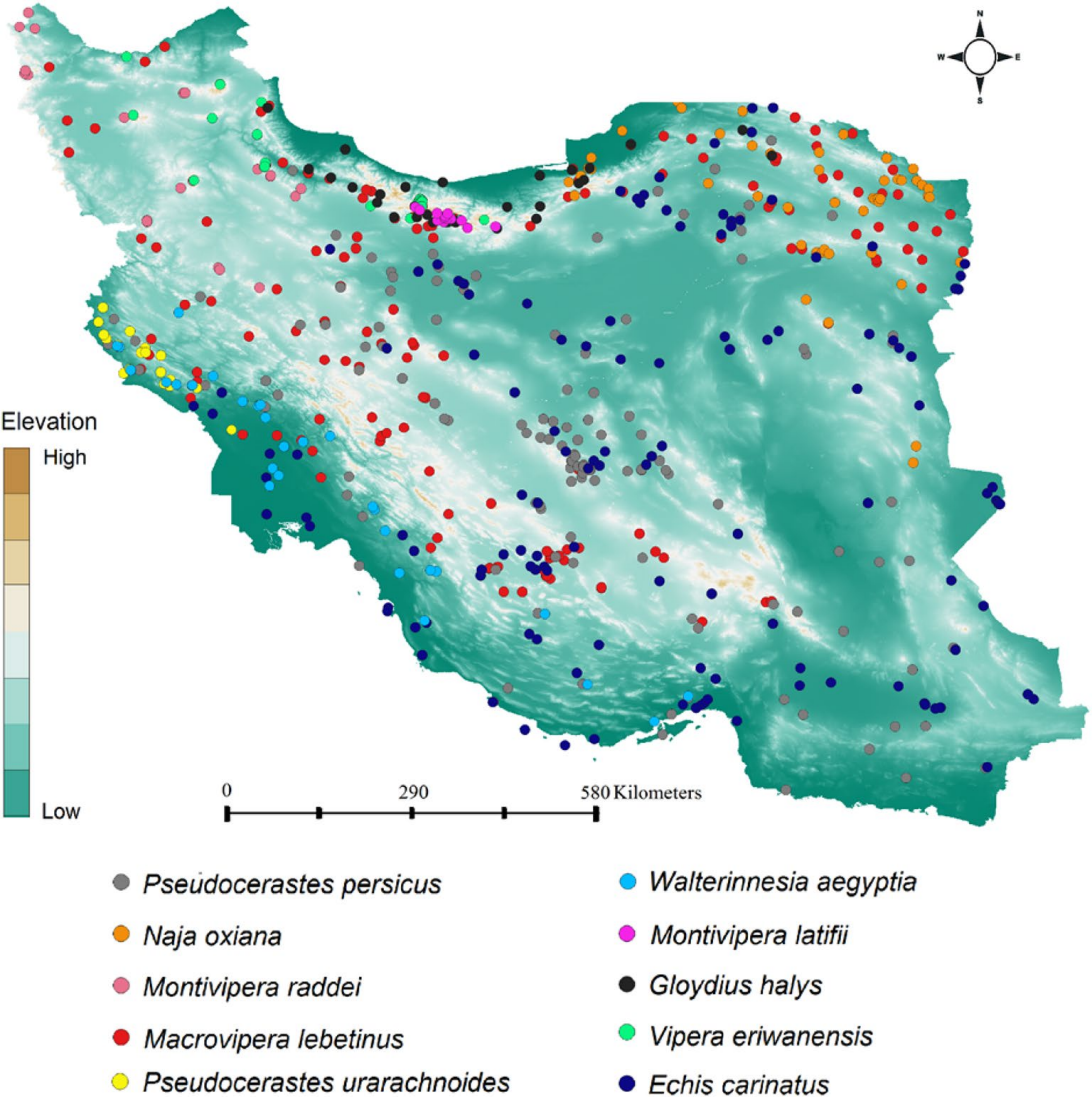
Distribution records of the 10 medically important venomous snakes (*Echis carinatus* (148 points), *Gloydius halys* (34 points), *Macrovipera lebetina* (150 points), *Montivipera latifii* (20 points), *Montivipera raddei* (23 points), *Naja oxiana* (51 points), *Pseudocerastes persicus* (151 points), *Pseudocerastes urarachnoides* (22 points), *Vipera eriwanensis* (54 points), *Walterinnesia aegyptia* (32 points)) on a topographic overview of Iran. Map was generated using QGIS 3.4.1 (https://www.qgis.org).

**Figure 8:**
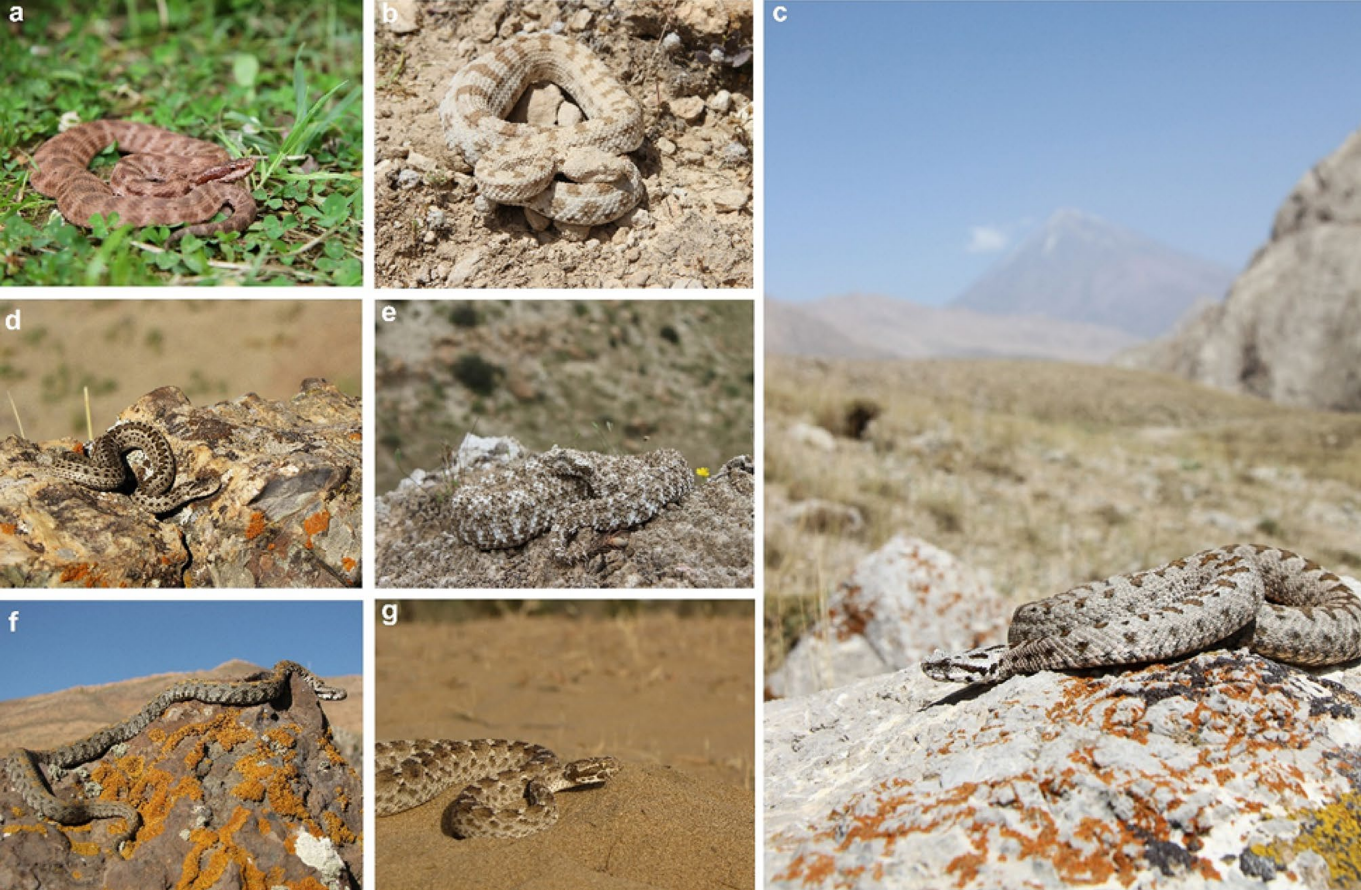
Photographs of medically important venomous snakes of Iran (*Gloydius halys* (**a**), *Pseudocerastes persicus* (**b**), *Montivipera latifii* (**c**), *Vipera eriwanensis* (**d**), *Pseudocerastes urarachnoides* (**e**), *Montivipera raddei* (**f**) and *Echis carinatus* (**g**)). All photos were taken in the species natural habitats. Photos by Masoud Yousefi.

In this study, distribution records of medically important venomous snakes of Iran^31^ gathered from different sources as follows: fieldworks, online databases (GBIF, VertNet), published books and papers ranging from 2000 to 2022. After gathering distribution records from different sources, we removed duplicates and thinned distribution records to match with climatic layers’ resolution (1 km^2^). Considering some species like *M. kuhrangica*, *Bungarus sindnus*, *Cerastes gasperettii*, and *Eristicophis macmahoni* have restricted range^31^ and limited distribution records it was not possible to develop robust habitat suitability models for them thus they were removed from further analysis^31^. We considered *M. lebetina* and *M. razii* together as one taxonomic unit^31^. We found enough distribution records to build habitat suitability model for 10 medically important venomous snake species (*E. carinatus* (148 points), *G. halys* (34 points), *M. lebetina* (150 points), *M. latifii* (20 points), *M. raddei* (23 points), *N. oxiana* (51 points), *P. persicus* (151 points), *P. urarachnoides* (22 points), *V. eriwanensis* (54 points), *W. aegyptia* (32 points)) in Iran^31^ (Figs. 7 and 8).

### Predictor variables

For climate data, we used CHELSA high resolution climatologies version 2.1^48^.This platform provides downscaled climate data for current and future climate conditions. We downloaded the bioclimatic variables at a 30 arcsec (~ 1 km) resolution for current climate and future climate (years 2041–2070 and 2071–2100) scenarios from five different CMIP6 Global Circulation Models (GCMs) (GFDL-ESM4, IPSL-CM6A-LR, MPI-ESM1-2-HR, MR-ESM2-0, UKESM1-0-LL). CMIP6 GCMs are state-of-the-art and using several GCMs is reducing the uncertainty coming from the single GCMs. The selected scenarios are corresponding to the available data. Further, we used two (SSP126, SSP585) different shared socioeconomic pathways (SSPs) for each GCM to represent the uncertainty of future CO2 emissions and therefore the expected change in climate. As predictor variables for habitat suitability modelling, we selected four bioclimatic variables including growing degree days heat sum above 10 °C (GDD), temperature seasonality (Bio4), annual precipitation (Bio12) and precipitation of the driest quarter (Bio17). Bioclimatic variables are gridded layers containing information about the climate in a particular region. They are derived variables from the monthly min, max, mean temperature, and mean precipitation values and were developed for species distribution modeling and related ecological applications. Those variables have shown to be reliable predictors for SDMs in a multitude of studies. Growing degree days are the heat sum of all days above the 10 °C temperature accumulated over 1 year, temperature seasonality is defined as standard deviation of the monthly mean temperatures, annual precipitation is the accumulated precipitation amount over 1 year and precipitation of the driest quarter is defined as the accumulated precipitation within the driest quarter. The selection was a combination of choosing species specific biologically relevant predictors and removing autocorrelated variables^13,34,38^. Additionally, we added slope as a fifth predictor variable. Slope was calculated from the Shuttle Radar Topography Mission (SRTM) digital elevation model^49^ at a 30 arcsec (~ 1 km) resolution with the *terrain* function of the *raster* package (version 3.4–13^50^). We included slope because it is an important predictor for habitat suitability of snakes. While climatic variables change over time, slope remains constant. However, future habitat suitability will still depend highly on slope. For instance, species occurring in flat areas will not adapt to terrain with steep slopes, even though the climate might become more suitable in the steep slopes. Including this variable thus is important to not overestimate the magnitude of change. For the SDM predictor variables, we chose biologically relevant variables and removed some correlated variables during this process as described in the methods section. We started with all bioclimatic variables and reduced the number of predictors in an empirical process of adding and removing variables to the SDMs in order to improve their performance. As all bioclimatic variables are derived from temperature and precipitation, some of those variables are highly correlated (e.g. maximum temperature and temperature of the warmest month). Excluding auto-correlated variables can help to avoid violating the assumption of independence of data points. Therefore, it is important to reduce the number of variables and select those which are less correlated and therefore improve the accuracy and reliability of the SDM.

### Modeling snakebite risk under current and future climate

For modelling the suitable habitat of the snake species under current and future climate, we used an equally weighted ensemble approach of five different habitat suitability modeling algorithms (HSM algorithms). We used GLMs (*base* R-package; R environment^51^), GAMs (*gam* R-package version 1.20.1^52^), GBMs (*gbm* R-packge version 2.1.8^53^), RandomForests (*randomForest* R-package version 2.1.8^54^) and Maxent (*dismo* R-package version 1.3-5^55^). For each species, we generated 5000 pseudo-absences by randomly sampling coordinates from the ecoregions where the species occurred and down- weighted the absences in the model algorithms to balance the presence-absence prevalence to 0.5^56^. To assess model performance, we used a split-sample approach (70% training data and 30% evaluation data) with 20 repetitions. The total number of points was 685 in which 480 points (70%) were used for as training data. Performance was measured using the area under the receiver operating characteristic curve (AUC)^57,58^ and True Skill Statistics (TSS)^59^. TSS values range from − 1 to + 1, where + 1 indicates perfect performance and value of zero meaning random predictions. AUC values range from 0 to 1, a value of 0.5 indicates that the performance of the model is not better than random, while values closer to 1.0 indicate better model performance^57–59^. We additionally checked the projections of the models visually (expert-based evaluation). Binary classifications were done using the sensitivity–specificity sum maximization approach (R package *presenceAbsence* 1.1.9^60^). Model variable importance was assessed using the *variables_importance* function of the *biomod2* package (version 3.5.1^61^) for GLMs, GAMs and GBMs, the *importance* function of the *randomForest* package (version 2.1.8^54^) for RandomForest models and the ecospat.*maxentvarimport* function of the *ecospat* package (version 3.2^62^) for Maxent models. We evaluated the importance of each variable across the different algorithms to show the variation in variable importance.

### Mapping snakebite hotspot, community composition changes and vulnerability to snakebite

To map the snakebite hotspot, we calculated the species richness of the 10 medically important venomous snakes by stacking binary habitat suitability maps of all species and calculating the sum for each grid cell in R^51^. For the community composition analysis, species richness maps were converted to matrices and then compared with the *ecospat.CommunityEval* function of the *ecospat* package (version 3.2^62^). Finally, to map vulnerability to snakebite across the country we considered two factors a) distance to cities which can provide primary health care services (accessibility to health care) and b) exposure to medically important venomous snakes (binary habitat suitability maps). We calculated a distance-to-city layer as the distance of each grid cell to the nearest city using the *distance* function of the *raster* package (version 3.4-13^50^). For each species we masked the binary habitat suitability map with the distance-to-city layer to get a vulnerability to snakebite map for each medically important venomous snake. For the final risk maps, we multiplied the species richness maps with the distance-to-city layer. From this we extracted the values from the vulnerability to snakebite maps for each cell to get the vulnerability index.

## Supplementary Information

Supplementary Figures.

## Data Availability

The datasets generated and analysed during the current study are available from the sources described in the manuscript and also the corresponding author on reasonable request.
