# Supplementary material for: Identifying high snakebite risk area under climate change for community education and antivenom distribution
Source: Sci Rep. 2023 May 20;13:8191. doi: 10.1038/s41598-023-35314-1 (PMC10199932; doi:10.1038/s41598-023-35314-1)
Supplement: Supplementary file 1 — Supplementary Figures. [file 41598_2023_35314_MOESM1_ESM.pdf]

Identifying high snakebite risk area under climate change for community education and antivenom distribution

Masoud Yousefi, Saeed Hosseinian Yousefkhani, Marc Grünig, Anooshe Kafash, Mahdi Rajabizadeh, Eskandar Rastegar Pouyani

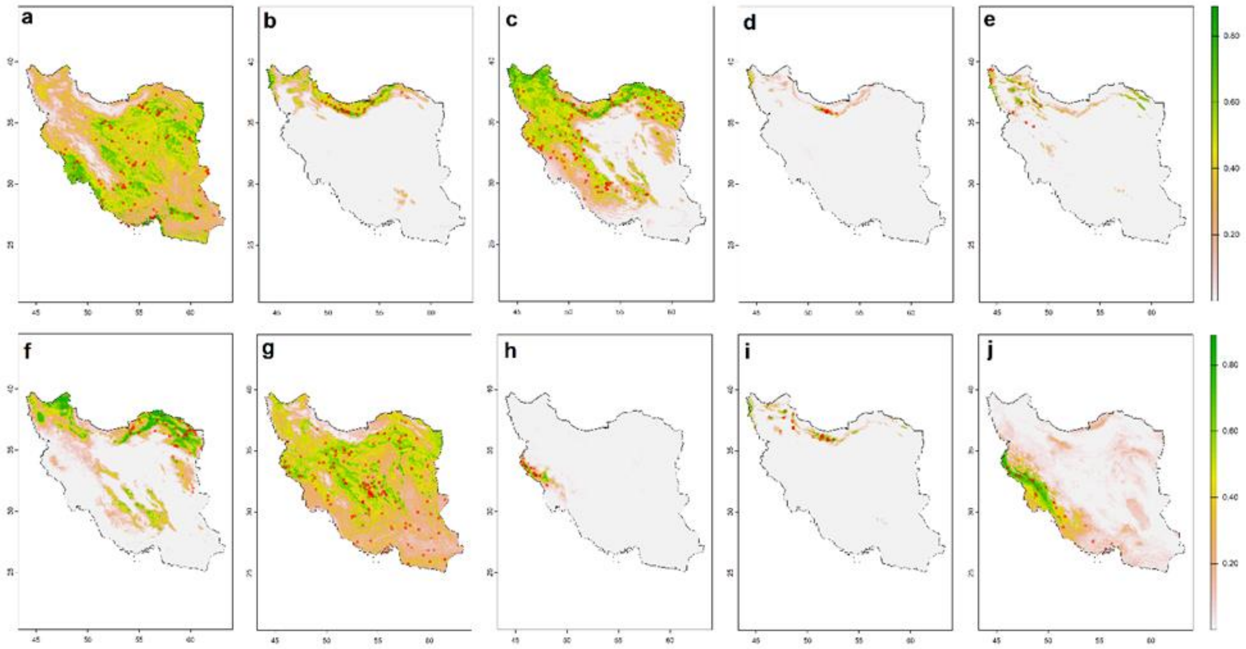

Figure S1. Habitat suitability maps of the 10 species (*Echis carinatus* (a), *Gloydius halys* (b), *Macrovipera lebetina* (c), *Montivipera latifii* (d), *Montivipera raddei* (e), *Naja oxiana* (f), *Pseudocerastes persicus* (g), *Pseudocerastes urarachnoides* (h), *Vipera eriwanensis* (i), *Walterinnesia aegyptia* (j)) under future climate (years 2041-2070 under SSP126). Models were generated and mapped in R 4.0.2 (https://cran.r-project.org/).

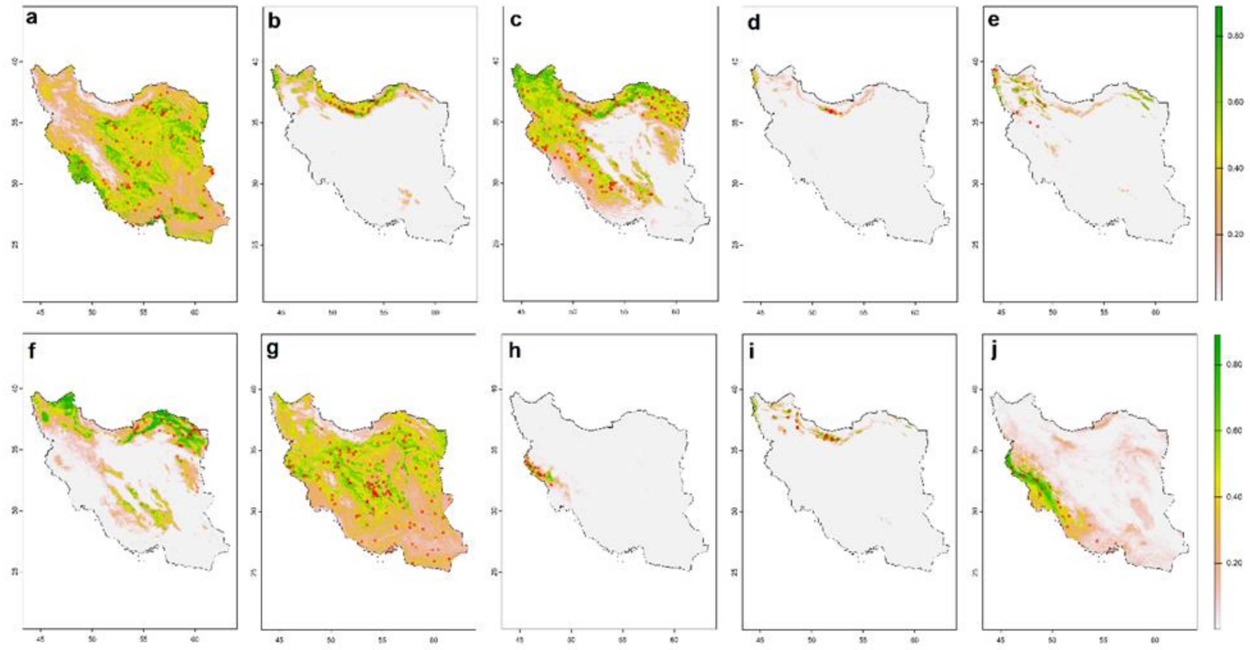

Figure S2. Habitat suitability maps of the 10 species (*Echis carinatus* (a), *Gloydius halys* (b), *Macrovipera lebetina* (c), *Montivipera latifii* (d), *Montivipera raddei* (e), *Naja oxiana* (f), *Pseudocerastes persicus* (g), *Pseudocerastes urarachnoides* (h), *Vipera eriwanensis* (i), *Walterinnesia aegyptia* (j)) under future climate (year 2041-2070 under SSP585). Models were generated and mapped in R 4.0.2 (https://cran.r-project.org/).

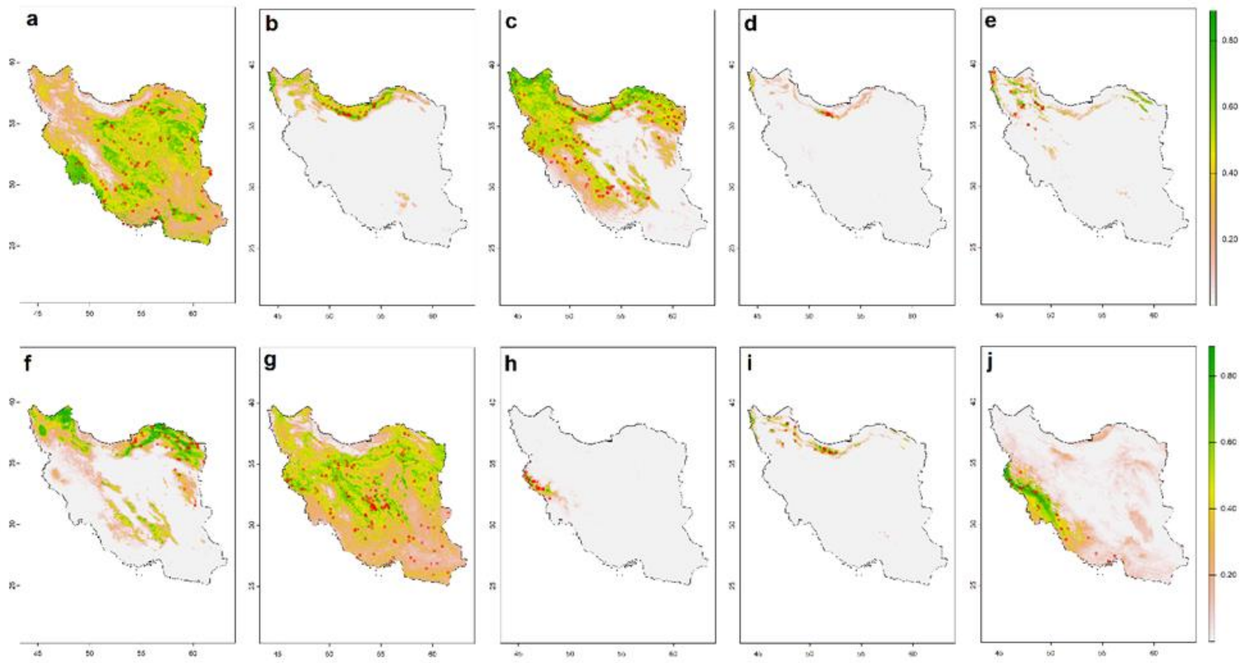

Figure S3. Habitat suitability maps of the 10 species (*Echis carinatus* (a), *Gloydius halys* (b), *Macrovipera lebetina* (c), *Montivipera latifii* (d), *Montivipera raddei* (e), *Naja oxiana* (f), *Pseudocerastes persicus* (g), *Pseudocerastes urarachnoides* (h), *Vipera eriwanensis* (i), *Walterinnesia aegyptia* (j)) under future climate (year 2071-2100 under SSP126). Models were generated and mapped in R 4.0.2 (https://cran.r-project.org/).
